# Supplementary material for: Effects of oxidative stress on hepatic encephalopathy pathogenesis in mice
Source: Nat Commun. 2023 Jul 24;14:4456. doi: 10.1038/s41467-023-40081-8 (PMC10366183; doi:10.1038/s41467-023-40081-8)
Supplement: Supplementary file 1 — Supplementary Information [file 41467_2023_40081_MOESM1_ESM.pdf]

Supplementary Information

Effects of oxidative stress on hepatic encephalopathy pathogenesis in mice

Yunhu Bai[1,2,†], Kenan Li[3,†], Xiaodong Li[1], Xiyu Chen[1], Jie Zheng[3], Feifei Wu[3], Jinghao Chen[1], Ze Li[3], Shuai Zhang[3], Kun Wu[4], Yong Chen[1,*], Yayun Wang[3,*], Yanling Yang[1,*]

[1] Department of Hepatobiliary Surgery, Xi-Jing Hospital, The Fourth Military Medical University, Xi'an, 710032, China

[2] Department of General Surgery, 988 Hospital of Joint Logistic Support Force, Zheng Zhou, 450000, China

[3] Specific Lab for Mitochondrial Plasticity Underlying Nervous System Diseases, National Demonstration Center for Experimental Preclinical Medicine Education, The Fourth Military Medical University, Xi'an, 710032, China

[4] Department of pharmacy, 518 Hospital, Xi'an, 710032, China

† These authors contributed to this work equally.

[*] **Corresponding authors:**

Yanling Yang; Email: yangyanl@fmmu.edu.cn;

Yayun Wang; Email: wangyy@fmmu.edu.cn;

Yong Chen; Email: gdwkcy@163.com;

Supplementary Figures

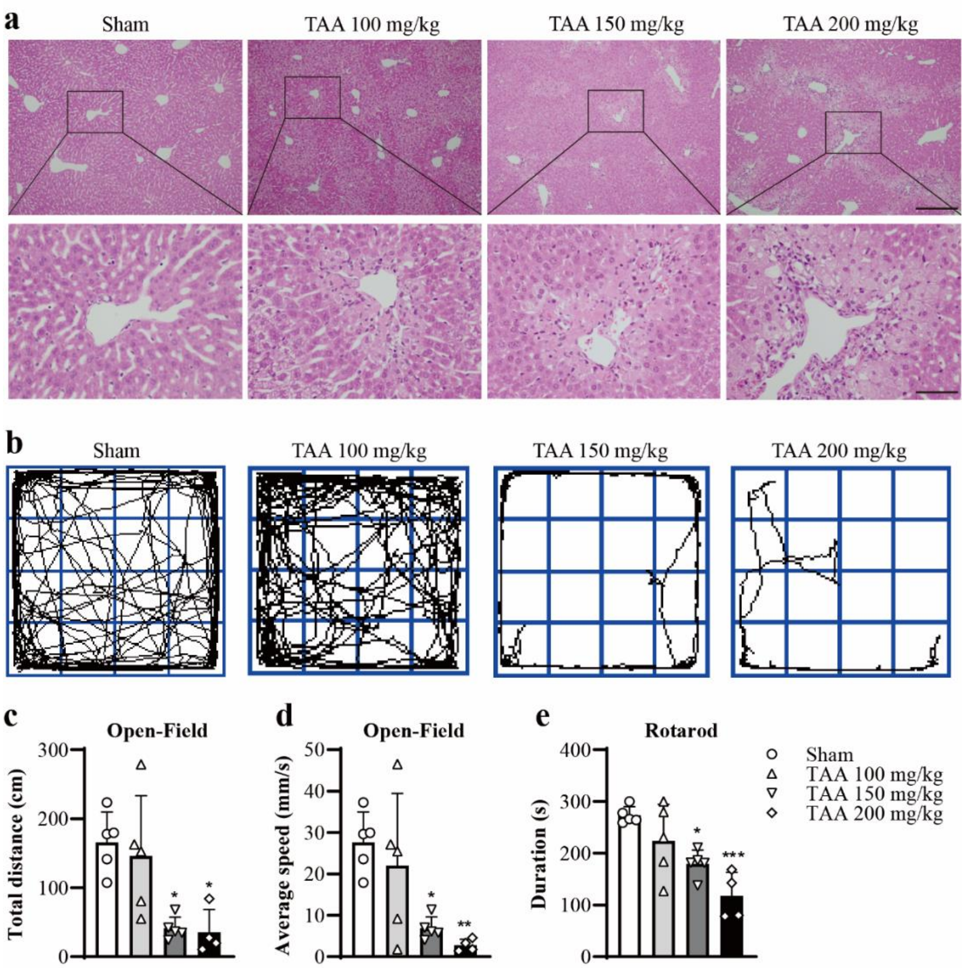

Supplementary Fig. 1 Effects of different doses of TAA on liver injury and motor function in mice. (a) H&E staining of the liver cell damage caused by different doses of TAA (Bar = 100 μm (top), Bar = 30 μm (bottom)). (b) Schematic traces of the open field tests in different groups. (c and d) Analysis of the total distance and average speed in the open field test (Sham, TAA 100 mg/kg, TAA 150 mg/kg n = 5, TAA 200 mg/kg, n = 4). (e) Analysis of duration in the rotarod test (Sham, TAA 100 mg/kg, TAA 150 mg/kg n = 5, TAA 200 mg/kg, n = 4). Data are presented as mean ± SD. * *P < 0.05,* ** *P < 0.01,* *** *P < 0.001 vs* Sham. One-way ANOVA with LSD's multiple comparison tests for c, d, e. Source data are provided as a Source Data file.

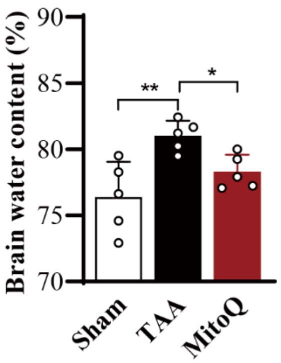

Supplementary Fig. 2 MitoQ alleviated TAA-induced cerebral edema (n = 5). Data are presented as mean ± SD. * $P < 0.05$, ** $P < 0.01$ *vs* Sham. One-way ANOVA with LSD's multiple comparison tests was used. Source data are provided as a Source Data file.

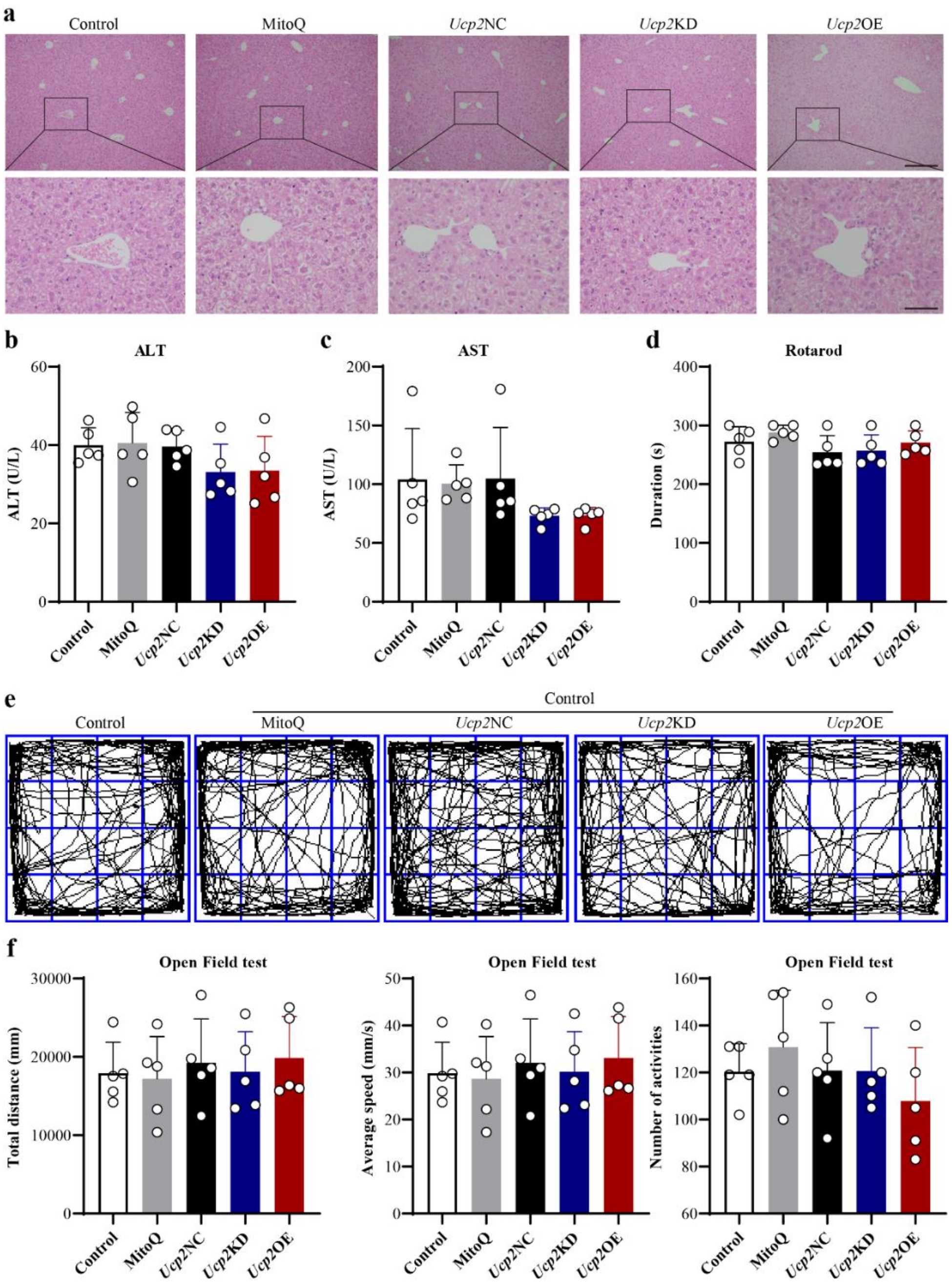

Supplementary Fig. 3 MitoQ and *Ucp2* adeno-associated viruses had no effect on liver function and motor performance in normal mice. (a) Represent images of H&E staining in normal control group, MitoQ - treated normal mice group (MitoQ), *Ucp2* NC virus treated normal mice group (*Ucp2* NC), *Ucp2* KD virus - treated normal mice group (*Ucp2* KD), *Ucp2* OE virus - treated normal mice group (*Ucp2* OE). Bar = 100 μm on the top and Bar = 30 μm on the bottom. (b and c) The levels of ALT (b) and AST (c) in different groups (n = 5). (d) The analysis of rotarod test in different groups (n = 5). (e)

Representative schematic traces of open field tests in different groups. (f) The analysis of the total distance, average speed, and the number of activities of open field tests in different groups (n = 5). Data are presented as mean ± SD. One-way ANOVA with LSD's multiple comparison tests for all data. Source data are provided as a Source Data file.

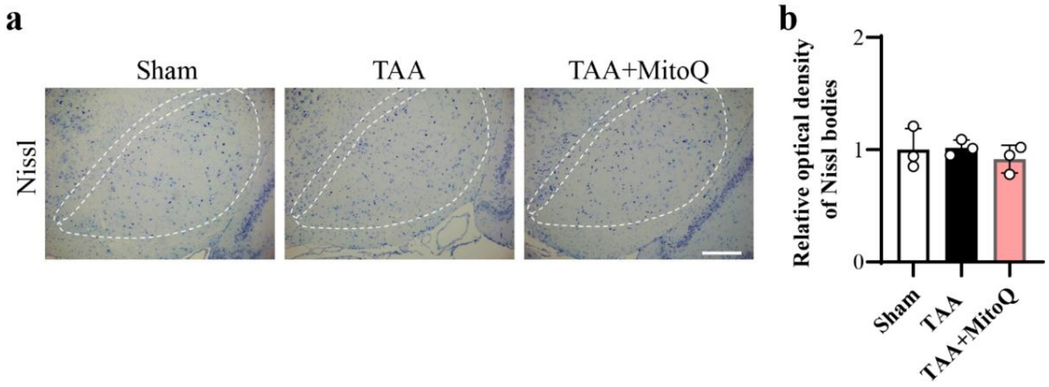

Supplementary Fig. 4 Effect of MitoQ on the number of SNr neurons. (a) Representative pictures of nissle staining of SNr in sham group, TAA + saline group and TAA + MitoQ groups. (b) Quantitative analysis of the number of SNr neurons in different groups (n = 3). Data are presented as mean ± SD. One-way ANOVA with LSD's multiple comparison tests for b. Source data are provided as a Source Data file.

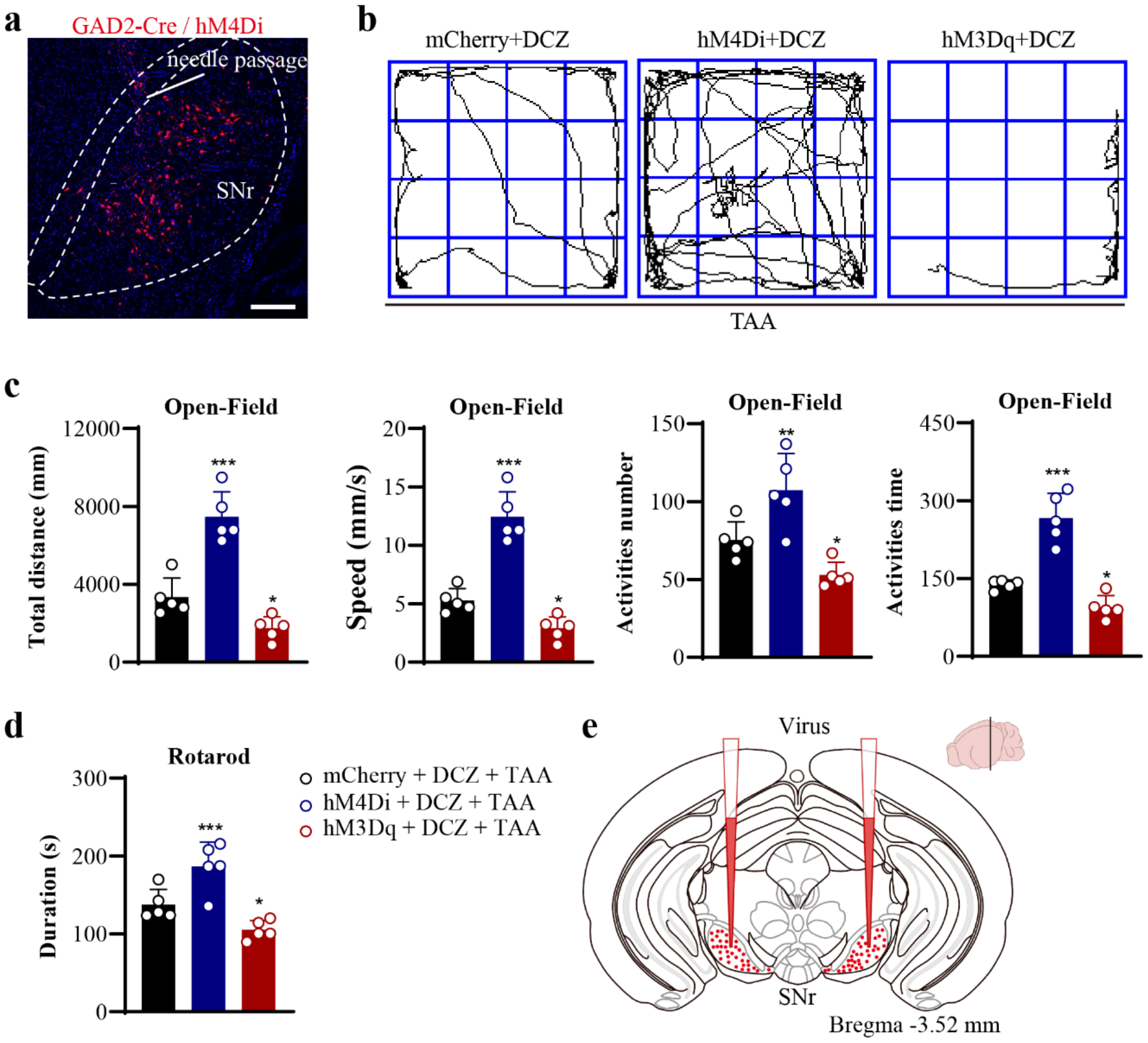

Supplementary Fig. 5 Causal effects of SNr^{Gad2} neuron activity on motor behavior in MHE. (a) Representative confocal images to confirm the correct location of hM4Di virus (mCherry) at SNr. (b) Schematic traces of the open field tests in control group (mcherry + DCZ), hM4Di - treated group (hM4Di + DCZ) and hM3Dq - treated group (hM3Dq + DCZ), activated by i.p. injection of DCZ. (c) The analysis of the total distance, average speed, activity numbers, and activity time of the open field test in different groups (n = 5). (d) The analysis of duration of the rotarod test in different groups (n = 5). (e) Schematic drawing showing the mice receiving virus injection within the SNr. Data are presented as mean ± SD. * *P < 0.05, *** P < 0.01, *** *P < 0.001 vs* mCherry + DCZ. One-way ANOVA with LSD's multiple comparison tests for all data. Source data are provided as a Source Data file.

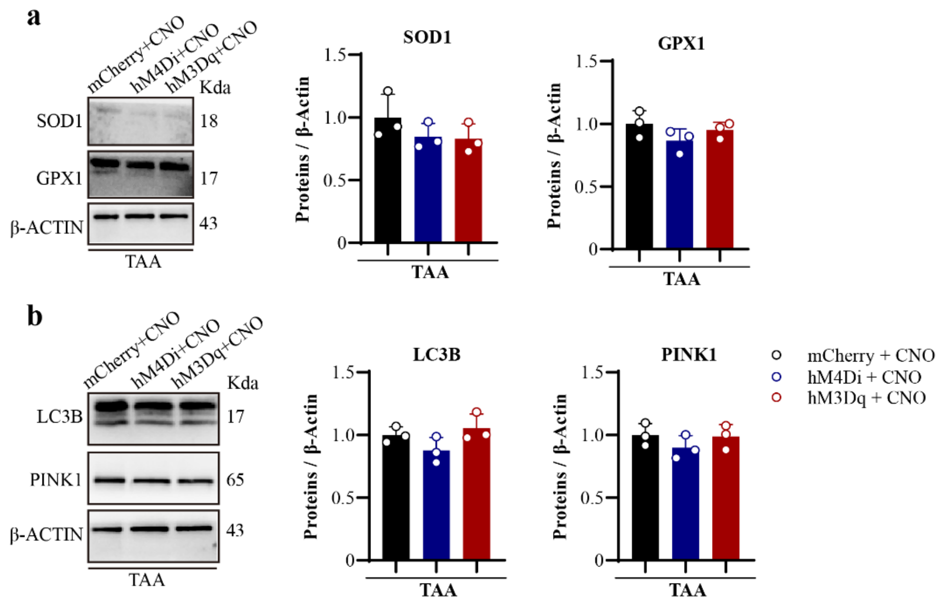

Supplementary Fig. 6 Effect of DREADDs on the antioxidative and autophagy markers. (a) Western blot results and statistical analysis of SOD1 and GPX1 level in SNr of control (mCherry + CNO), chemogenetic inhibition (Gi + CNO), and chemogenetic activation (Gq + CNO) groups (n = 3). (b) Western blot results and statistical analysis of LC3B and PINK1 level in SNr of mCherry + CNO, Gi + CNO, and Gq + CNO groups (n = 3). Data are presented as mean ± SD. One-way ANOVA with LSD's multiple comparison tests for all data. Source data are provided as a Source Data file.

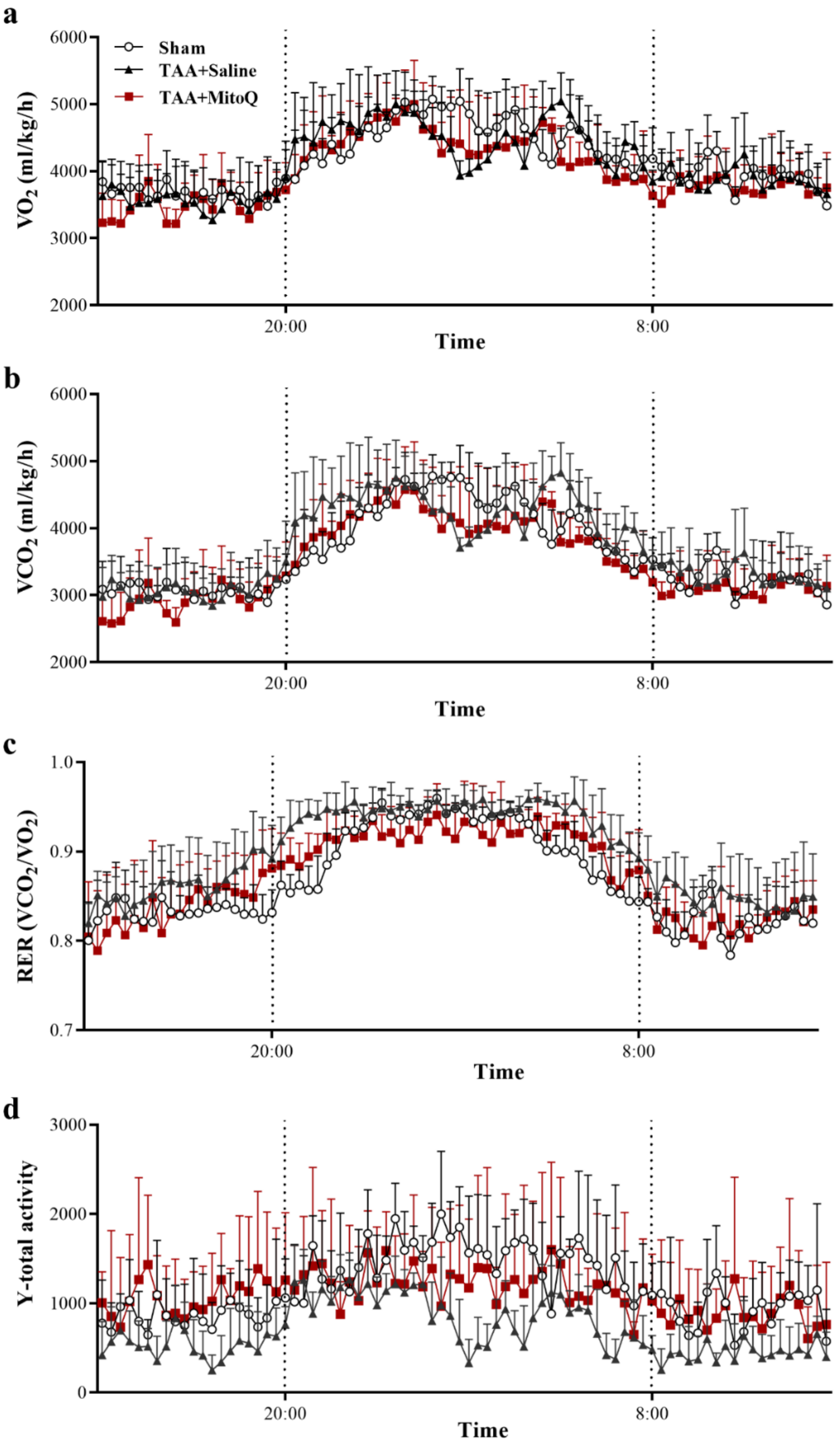

Supplementary Fig. 7 Effect of MitoQ on basal metabolic rate in mice. (a - c) VO_2 (a), VCO_2 (b), RER (c) of test period in Sham, TAA + Saline, and TAA + MitoQ groups (n = 5). (d) The Y-total activity of the test period in Sham, TAA + Saline, and TAA + MitoQ

groups (n = 5). Data are presented as mean ± SD. VO_2: Oxygen consumption, VCO_2: Carbon dioxide production, RER: Respiratory exchange ratio. Source data are provided as a Source Data file.

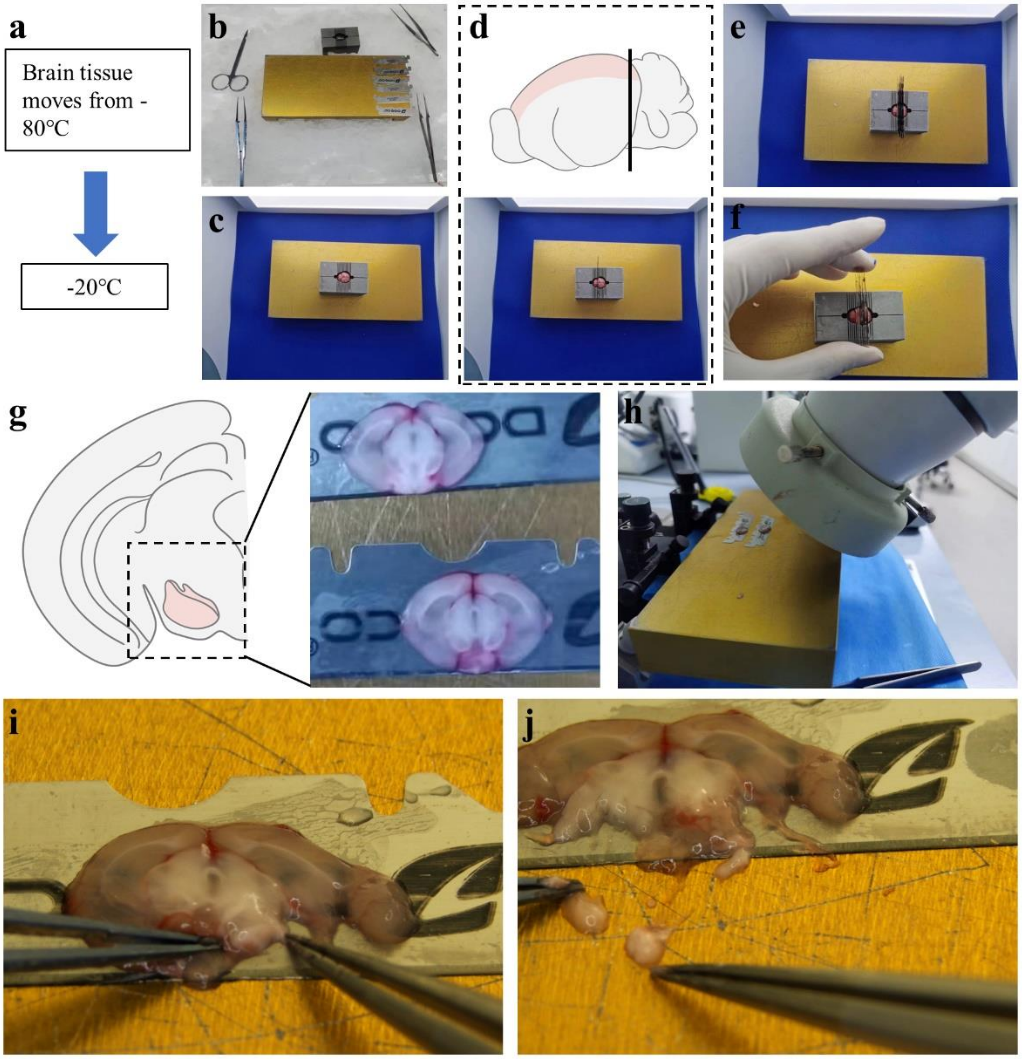

Supplementary Fig. 8 Isolation of SNr. (a) The brain tissue was removed from -80 °C to -20 °C. (b) Instruments were pre-cooled before sampling and kept at a low temperature throughout the process. (c) The brain tissue was removed from -20 °C and placed in the mouse brain mold. (d) The brain was set with dorsal side up to define the cerebellar boundary line with the rostrocaudal axis. (e) 5 blades were placed in a row to the ventral side of the brain. (f) The blade was aligned with the thumb and middle finger and pressed down with the forefinger for vertical and quick cutting. (g) Brain slices were separated. (h) Brain slices were transferred to be under the stereomicroscopy. (i and j) Microsurgery forceps were used to dissociate bilateral SNr area. Source data are provided as a Source Data file.

Supplementary statistical data

Fig. 1

Two-tailed, unpaired, Student’s *t*-test for all data in Fig. 1.
Fig. 1b – ALT, $P = 0.003$, $t = 3.707$, df = 12.
Fig. 1c – AST, $P < 0.001$, $t = 7.697$, df = 12.
Fig. 1d – Tbil, $P < 0.001$, $t = 6.545$, df = 12.
Fig. 1e - blood ammonia, $P < 0.001$, $t = 12.370$, df = 10.
Fig. 1f – brain ammonia, $P < 0.001$, $t = 7.276$, df = 10.
Fig. 1g – SOD, $P < 0.001$, $t = 6.657$, df = 10.
Fig. 1h – GPx, $P = 0.009$, $t = 3.250$, df = 10.
Fig. 1i – Rotarod, $P < 0.001$, $t = 7.108$, df =16.
Fig. 1k – OFT Total distance, $P < 0.001$, $t = 9.744$, df = 16.
OFT - Average speed, $P < 0.001$, $t = 9.741$, df = 16.

Fig. 2

Two-tailed, unpaired, Student’s *t*-test for all data in Fig. 2.
SNr：
Fig. 2b
LC-3B, $P = 0.002$, $t = 7.472$, df = 4.
PINK1, $P = 0.007$, $t = 5.100$, df = 4.
SOD1, $P = 0.011$, $t = 4.520$, df = 4.
GPX1, $P = 0.042$, $t = 2.956$, df = 4.
UCP2, $P = 0.013$, $t = 4.315$, df = 4.

Fig. 2d
UCP5, $P = 0.087$, $t = 2.262$, df = 4.
UCP4, $P = 0.345$, $t = 1.069$, df = 4.

Fig. 2f
DRP1, $P = 0.400$, $t = 0.9428$, df = 4.
p-DRP1 Ser616, $P = 0.006$, $t = 5.226$, df = 4.
MFN2, $P = 0.964$, $t = 0.04796$, df = 4.
MFF, $P = 0.003$, $t = 6.470$, df = 4.
FIS1, $P = 0.006$, $t = 5.252$, df = 4.

Fig. 2i
Individual, $P < 0.001$, $t = 6.124$, df = 6.

Cortex
Fig. 2k:
UCP5, $P = 0.089$, $t = 2.239$, df = 4.
UCP4, $P = 0.169$, $t =1.679$, df = 4.
UCP2, $P = 0.313$, $t =1.153$, df = 4.
SOD1, $P < 0.001$, $t = 9.393$, df = 4.
GPX1, $P = 0.506$, $t = 0.7289$, df = 4.

Fig. 2m
LC-3B, $P = 0.003$, $t = 6.704$, df = 4.
PINK1, $P = 0.005$, $t = 5.665$, df = 4.

Fig. 2o
DRP1, $P = 0.069$, $t = 2.468$, df = 4.
p-DRP1 Ser616, $P = 0.340$, $t = 1.082$, df = 4.
MFN2, $P = 0.569$, $t = 0.6202$, df = 4.
MFF, $P = 0.340$, $t = 1.081$, df = 4.
FIS1, $P = 0.115$, $t = 2.011$, df = 4.

Fig. 3

Two-tailed, unpaired, Student's *t*-test for all data except one-way ANOVA with LSD's multiple comparison tests for l.

c – ALT, $P = 0.004$, $t = 3.681$, df = 10.
d - AST, $P < 0.001$, $t = 6.333$, df = 10.
e - blood ammonia, $P < 0.001$, $t = 6.411$, df = 10.
f - Brain ammonia, $P = 0.020$, t =2.763, df = 10.
g – SOD, $P = 0.003$, t =3 .709, df = 11.
h – GPx, $P = 0.002$, $t = 4.205$, df = 11.
i: CatWalk-Run time, $P = 0.004$, $t = 3.528$, df = 12.
CatWalk-Speed, $P < 0.001$, $t = 8.452$, df = 12.
j – Rotarod, $P = 0.002$, $t = 3.871$, df = 12.
k:
OFT-Total distance, $P < 0.001$, $t = 4.566$, df = 15.
OFT-Average speed, $P < 0.001$, $t = 4.570$, df = 15.
l: One-way ANOVA with LSD's multiple comparison tests for l.

Statistical analysis of ROS staining in Sham, TAA + Saline, and TAA + MitoQ groups $F_{(2, 9)} = 13.729$, $P = 0.002$, Sham vs. TAA + Saline: $P < 0.001$, TAA + Saline vs. TAA + MitoQ: $P = 0.009$.

m:
UCP2, $P = 0.015$, $t = 4.064$, df = 4.
SOD1, $P = 0.024$, $t = 3.521$, df = 4.
GPX1, $P = 0.044$, $t = 2.895$, df = 4.
n
LC-3B, $P = 0.028$, $t = 3.393$, df = 4.
PINK1, $P = 0.002$, $t = 7.770$, df = 4.
o
DRP1, $P = 0.087$, $t = 2.262$, df = 4.
p-DRP1 Ser616, $P = 0.026$, $t = 3.438$, df = 4.
MFF, $P = 0.093$, $t = 2.196$, df = 4.
FIS1, $P = 0.042$, $t = 2.948$, df = 4.

Fig.4

One-way ANOVA with LSD's multiple comparison tests for all.

c – ALT: Statistical analysis of ALT in Sham, Sham + MitoQ, BDL and BDL + MitoQ groups $F(3, 20) = 37.990$, $P < 0.001$, Sham vs. BDL: $P < 0.001$, BDL vs. BDL + MitoQ: $P < 0.001$.

d - AST: Statistical analysis of AST in Sham, Sham + MitoQ, BDL and BDL + MitoQ groups $F(3, 20) = 21.791$, $P < 0.001$, Sham vs. BDL: $P < 0.001$, BDL vs. BDL + MitoQ: $P = 0.012$.

e - TBil: Statistical analysis of TBil in Sham, Sham + MitoQ, BDL and BDL + MitoQ groups $F(3, 20) = 26.040$, $P < 0.001$, Sham vs. BDL: $P < 0.001$, BDL vs. BDL + MitoQ: $P = 0.386$.

f - blood ammonia: Statistical analysis of blood ammonia in Sham, Sham + MitoQ, BDL and BDL + MitoQ groups $F(3, 20) = 21.212$, $P < 0.001$, Sham vs. BDL: $P < 0.001$, BDL vs. BDL + MitoQ: $P = 0.003$.

g – Rotarod: Statistical analysis of latency time in Sham, Sham + MitoQ, BDL and BDL + MitoQ groups $F(3, 36) = 44.127$, $P < 0.001$, Sham vs. BDL: $P < 0.001$, BDL vs. BDL + MitoQ: $P = 0.024$.

i –

OFT-Total distance: Statistical analysis of total distance in Sham, Sham + MitoQ, BDL and BDL + MitoQ groups $F(3, 36) = 55.917$, $P < 0.001$, Sham vs. BDL: $P < 0.001$, BDL vs. BDL + MitoQ: $P = 0.031$.

OFT-Average speed: Statistical analysis of average speed in Sham, Sham + MitoQ, BDL and BDL + MitoQ groups $F(3, 36) = 55.918$, $P < 0.001$, Sham vs. BDL: $P < 0.001$, BDL vs. BDL + MitoQ: $P = 0.0312$.

OFT-Number of activities: Statistical analysis of number of activities in Sham, Sham + MitoQ, BDL and BDL + MitoQ groups $F(3, 36) = 39.521$, $P < 0.0001$, Sham vs. BDL: $P < 0.001$, BDL vs. BDL + MitoQ: $P < 0.001$.

k

UCP2: Statistical analysis of UCP2 in Sham, Sham + MitoQ, BDL and BDL + MitoQ groups $F(3, 8) = 19.015$, $P < 0.001$, Sham vs. BDL: $P < 0.001$, Sham + MitoQ vs. BDL + MitoQ: $P = 0.005$, BDL vs. BDL + MitoQ: $P = 0.021$.

SOD1: Statistical analysis of SOD1 in Sham, Sham + MitoQ, BDL and BDL + MitoQ groups $F(3, 8) = 8.940$, $P = 0.006$, Sham vs. BDL: $P = 0.004$, BDL vs. BDL + MitoQ: $P = 0.045$.

GPX1: Statistical analysis of GPX1 in Sham, Sham + MitoQ, BDL and BDL + MitoQ groups $F(3, 8) = 0.097$, $P = 0.960$, Sham vs. BDL: $P = 0.986$, BDL vs. BDL + MitoQ: $P = 0.761$.

m

LC-3B: Statistical analysis of LC-3B in Sham, Sham + MitoQ, BDL and BDL + MitoQ groups $F(3, 8) = 25.970$, $P < 0.001$, Sham vs. BDL: $P < 0.001$, BDL vs. BDL + MitoQ: $P < 0.001$.

PINK1: Statistical analysis of PINK1 in Sham, Sham + MitoQ, BDL and BDL + MitoQ groups $F(3, 8) = 8.6153$, $P = 0.007$, Sham vs. BDL: $P = 0.002$, BDL vs. BDL + MitoQ: $P = 0.008$.

o

DRP1:

Statistical analysis of DRP1 in Sham, Sham + MitoQ, BDL and BDL + MitoQ groups $F(3, 8) = 0.245$, $P = 0.863$, Sham vs. BDL: $P = 0.846$, BDL vs. BDL + MitoQ: $P = 0.599$.

p-DRP1 Ser616:
Statistical analysis of p-DRP1 Ser 616 in Sham, Sham + MitoQ, BDL and BDL + MitoQ groups F (3, 8) = 42.143, *P* < 0.001, Sham vs. BDL: *P* < 0.001, BDL vs. BDL + MitoQ: *P* < 0.001.
MFN2:
Statistical analysis of MFN2 in Sham, Sham + MitoQ, BDL and BDL + MitoQ groups F (3, 8) = 3.058, *P* = 0.092, Sham vs. BDL: *P* = 0.020, BDL vs. BDL + MitoQ: *P* = 0.081.
MFF:
Statistical analysis of MFF in Sham, Sham + MitoQ, BDL and BDL + MitoQ groups F (3, 8) = 34.960, *P* < 0.001, Sham vs. BDL: *P* < 0.001, BDL vs. BDL + MitoQ: *P* < 0.001.
FIS1:
Statistical analysis of FIS1 in Sham, Sham + MitoQ, BDL and BDL + MitoQ groups F (3, 8) = 5.527, *P* = 0.024, Sham vs. BDL: *P* = 0.008, BDL vs. BDL + MitoQ: *P* = 0.009.

Fig.5
Two-tailed, unpaired, Student's *t* -test for c and g.
c:
Total SNr, *P* = 0.033, *t* = 3.194, df = 4.
Medial SNr, *P* = 0.002, *t* = 7.399, df = 4.
Lateral SNr, *P* = 0.392, *t* = 0.958, df = 4.
g:
Total SNr, *P* = 0.002, *t* = 7.407, df = 4.
Medial SNr, *P* = 0.003, *t* = 6.245, df = 4.
Lateral SNr, *P* < 0.001, *t* = 10.590, df = 4.

Fig.6
Two-tailed, unpaired, Student's *t* -test for d and i
Two-tailed, paired, Student's *t* -test for f, g, k and l.
One-way ANOVA with LSD's multiple comparison tests for m.
d: Firing rate, *P* = 0.0123, *t* = 3.513, df = 6, n = 4.
f:
OFT-Total distance
Saline-CNO-Pre vs. Saline-CNO-Post, *P* = 0.554, *t* = 0.644, df = 4, Number of pairs = 5.
TAA-CNO-Pre vs. TAA-CNO-Post, *P* < 0.001, *t* = 6.313, df = 6, Number of pairs = 7.
OFT-Average speed
Saline-CNO-Pre vs. Saline-CNO-Post, *P* = 0.643, *t* = 0.500, df = 4, Number of pairs = 5.
TAA-CNO-Pre vs. TAA-CNO-Post, *P* < 0.001, *t* = 6.330, df = 6, Number of pairs = 7.
g-Rotarod
Saline-CNO-Pre vs. Saline-CNO-Post, *P* = 0.7053, *t* = 0.4064, df = 4, Number of pairs = 5.
TAA-CNO-Pre vs. TAA-CNO-Post, *P* = 0.0483, *t* = 2.473, df = 6, Number of pairs = 7.
i -Firing rate, *P* = 0.0098, t = 3.723, df = 6.
k:
OFT-Total distance:

Saline-CNO-Pre vs. Saline-CNO-Post, $P = 0.112$, $t = 2.031$, df = 4, Number of pairs = 5
TAA-CNO-Pre vs. TAA-CNO-Post, $P = 0.008$, $t = 3.872$, df = 6, Number of pairs = 7.
OFT-Average speed:
Saline-CNO-Pre vs. Saline-CNO-Post, $P = 0.111$, $t = 2.036$, df = 4, Number of pairs = 5
TAA-CNO-Pre vs. TAA-CNO-Post, $P = 0.008$, $t = 3.934$, df = 6, Number of pairs = 7.
l-Rotarod:
Saline-CNO-Pre vs. Saline-CNO-Post, $P = 0.4134$, $t = 0.9120$, df = 4, Number of pairs = 5.
TAA-CNO-Pre vs. TAA-CNO-Post, $P = 0.002$, $t = 5.544$, df = 6, Number of pairs = 7.
m
UCP2:
One-way ANOVA with LSD's multiple comparison tests for all.
Statistical analysis of UCP2 in mCherry + CNO, Gi + CNO and Gq + CNO groups $F (2, 6) = 50.728$, $P < 0.001$, mCherry + CNO vs. Gi + CNO: $P = 0.001$, mCherry + CNO vs. Gq + CNO: $P = 0.007$.

Fig.7

One-way ANOVA with LSD's multiple comparison tests for all data.
b - UCP2: Statistical analysis of UCP2 level in TAA + *Ucp2* NC, TAA + *Ucp2* KD, and TAA + *Ucp2* OE groups $F (2, 6) = 37.0652$, $P = 0.0004$, TAA + *Ucp2*N C vs. TAA + *Ucp2* KD: $P = 0.0069$, TAA + *Ucp2* NC vs. TAA + *Ucp2* OE: $P = 0.0038$.
d - OFT-Total distance;
Statistical analysis of total distance in TAA + *Ucp2* NC, TAA + *Ucp2* KD, and TAA + *Ucp2* OE groups $F (2, 19) =127.8593$, $P < 0.0001$, TAA + *Ucp2* NC vs. TAA + *Ucp2* KD: $P < 0.0001$, TAA + *Ucp2* NC vs. TAA + *Ucp2*OE: $P < 0.0001$.
OFT-Average speed:
Statistical analysis of average speed in TAA + *Ucp2* NC, TAA + *Ucp2* KD, and TAA + *Ucp2* OE groups $F (2, 19) = 128.0687$, $P < 0.0001$, TAA + *Ucp2* NC vs. TAA + *Ucp2* KD: $P < 0.0001$, TAA + *Ucp2* NC vs. TAA + *Ucp2*OE: $P < 0.0001$.
f
SOD1
Statistical analysis of SOD1 level in TAA + *Ucp2* NC, TAA + *Ucp2* KD, and TAA + *Ucp2* OE groups $F (2, 6) =229.7078$, $P < 0.0001$, TAA + *Ucp2* NC vs. TAA + *Ucp2* KD: $P = 0.0003$, TAA + *Ucp2* NC vs. TAA + *Ucp2* OE: $P < 0.0001$.
GPX1
Statistical analysis of GPX1 level in TAA + *Ucp2* NC, TAA + *Ucp2* KD, and TAA + *Ucp2* OE groups $F (2, 6) = 159.2336$, $P < 0.0001$, TAA + *Ucp2* NC vs. TAA + *Ucp2* KD: $P = 0.0002$, TAA + *Ucp2* NC vs. TAA + *Ucp2* OE: $P < 0.0001$.

h
LC-3B
Statistical analysis of LC-3B level in TAA + *Ucp2* NC, TAA + *Ucp2* KD, and TAA + *Ucp2* OE groups $F (2, 6) = 23.6822$, $P = 0.0014$, TAA + *Ucp2* NC vs. TAA + *Ucp2* KD: $P = 0.0058$, TAA + *Ucp2* NC vs. TAA + *Ucp2*OE: $P = 0.0383$.
PINK1

Statistical analysis of PINK1 level in TAA + *Ucp2* NC, TAA + *Ucp2* KD, and TAA + *Ucp2* OE groups F (2, 6) = 68.9792, *P* < 0.0001, TAA + *Ucp2* NC vs. TAA + *Ucp2* KD: *P* = 0.0004, TAA + *Ucp2* NC vs. TAA + *Ucp2* OE: *P* = 0.0045.

j

p-DRP1 Ser616/: DRP1

Statistical analysis of p-DRP1 Ser616/ DRP1 level in TAA + *Ucp2* NC, TAA + *Ucp2* KD, and TAA + *Ucp2* OE groups F (2, 6) = 27.0952, *P* = 0.00010, TAA + *Ucp2* NC vs. TAA + *Ucp2* KD: *P* = 0.0031, TAA + *Ucp2* NC vs. TAA + *Ucp2*OE: *P* = 0.0480.

MFN2:

Statistical analysis of MFN2 level in TAA + *Ucp2* NC, TAA + *Ucp2* KD, and TAA + *Ucp2* OE groups F (2, 6) = 1.4234, *P* = 0.3120, TAA + *Ucp2* NC vs. TAA + *Ucp2* KD: *P* = 0.9272, TAA + *Ucp2* NC vs. TAA + *Ucp2* OE: *P* = 0.1826.

MFF:

Statistical analysis of MFF level in TAA + *Ucp2* NC, TAA + *Ucp2* KD, and TAA + *Ucp2* OE groups F (2, 6) = 0.5140, *P* = 0.8605, TAA + *Ucp2* NC vs. TAA + *Ucp2* KD: *P* = 0.9259, TAA + *Ucp2* NC vs. TAA + *Ucp2* OE: *P* = 0.6858.

FIS1

Statistical analysis of FIS1 level in TAA + *Ucp2* NC, TAA + *Ucp2* KD, and TAA + *Ucp2* OE groups F (2, 6) = 63.7652, *P* < 0.0001, TAA + *Ucp2* NC vs. TAA + *Ucp2* KD: *P* = 0.0002, TAA + *Ucp2* NC vs. TAA + *Ucp2* OE: *P* = 0.0224.

Fig.8

One-way ANOVA with LSD's multiple comparison tests for all data.

a

VO_2

Statistical analysis of VO_2 dark in Sham, TAA + Saline, and TAA + MitoQ groups F (2, 12) = 0.397, *P* = 0.6807, Sham vs. TAA + Saline: *P* = 0.964, TAA + Saline vs. TAA + MitoQ: *P* = 0.443.

Statistical analysis of VO_2 light in Sham, TAA + Saline, and TAA + MitoQ groups F (2, 12) = 0.912, *P* = 0.428, Sham vs. TAA + Saline: *P* = 0.537, TAA + Saline vs. TAA + MitoQ: *P* = 0.488.

Statistical analysis of VO_2 total in Sham, TAA + Saline, and TAA + MitoQ groups F (2, 12) = 0.574, *P* = 0.578, Sham vs. TAA + Saline: *P* = 0.818, TAA + Saline vs. TAA + MitoQ: *P* = 0.446.

b VCO_2

Statistical analysis of VCO_2 dark in Sham, TAA + Saline, and TAA + MitoQ groups F (2, 12) = 0.891, *P* = 0.436, Sham vs. TAA + Saline: *P* = 0.472, TAA + Saline vs. TAA + MitoQ: *P* = 0.208.

Statistical analysis of VCO_2 light in Sham, TAA + Saline, and TAA + MitoQ groups F (2, 12) = 1.192, *P* = 0.337, Sham vs. TAA + Saline: *P* = 0.758, TAA + Saline vs. TAA + MitoQ: *P* = 0.168.

Statistical analysis of VCO_2 total in Sham, TAA + Saline, and TAA + MitoQ groups F (2, 12) = 1.122, *P* = 0.358, Sham vs. TAA + Saline: *P* = 0.540, TAA + Saline vs. TAA + MitoQ: *P* = 0.162.

c RER

Statistical analysis of RER dark in Sham, TAA + Saline, and TAA + MitoQ groups $F (2, 12) = 7.762$, $P = 0.007$, Sham vs. TAA + Saline: $P = 0.003$, TAA + Saline vs. TAA + MitoQ: $P = 0.008$.
Statistical analysis of RER light in Sham, TAA + Saline, and TAA + MitoQ groups $F (2, 12) = 2.662$, $P = 0.111$, Sham vs. TAA + Saline: $P = 0.060$, TAA + Saline vs. TAA + MitoQ: $P = 0.081$.
Statistical analysis of RER total in Sham, TAA + Saline, and TAA + MitoQ groups $F (2, 12) = 7.560$, $P = 0.008$, Sham vs. TAA + Saline: $P = 0.004$, TAA + Saline vs. TAA + MitoQ: $P = 0.008$.

d Y-total activity

Statistical analysis of Y-total activity dark in Sham, TAA + Saline, and TAA + MitoQ groups $F (2, 12) = 3.697$, $P = 0.056$, Sham vs. TAA + Saline: $P = 0.020$, TAA + Saline vs. TAA + MitoQ: $P = 0.346$.
Statistical analysis of Y-total activity light in Sham, TAA + Saline, and TAA + MitoQ groups $F (2, 12) = 3.536$, $P = 0.062$, Sham vs. TAA + Saline: $P = 0.024$, TAA + Saline vs. TAA + MitoQ: $P = 0.093$.
Statistical analysis of Y-total activity total in Sham, TAA + Saline, and TAA + MitoQ groups $F (2, 12) = 3.919$, $P = 0.049$, Sham vs. TAA + Saline: $P = 0.016$, TAA + Saline vs. TAA + MitoQ: $P = 0.1802$.

e Boty temperature

Statistical analysis of body temperature 0 day in Sham, TAA + Saline, and TAA + MitoQ groups $F (2, 15) = 0.730$, $P = 0.499$, Sham vs. TAA + Saline: $P = 1.0$, Sham vs. TAA + MitoQ: $P = 0.312$, TAA + Saline vs. BDL + MitoQ: $P = 0.312$.
Statistical analysis of body temperature 1 day in Sham, TAA + Saline, and TAA + MitoQ groups $F (2, 15) = 2.205$, $P = 0.145$, Sham vs. TAA + Saline: $P = 0.082$, Sham vs. TAA + MitoQ: $P = 0.097$, TAA + Saline vs. BDL + MitoQ: $P = 0.929$.
Statistical analysis of body temperature 2 day in Sham, TAA + Saline, and TAA + MitoQ groups $F (2, 15) = 4.745$, $P = 0.025$, Sham vs. TAA + Saline: $P = 0.020$, Sham vs. TAA + MitoQ: $P = 0.016$, TAA + Saline vs. BDL + MitoQ: $P = 0.909$.
Statistical analysis of body temperature 3 day in Sham, TAA + Saline, and TAA + MitoQ groups $F (2, 15) = 7.078$, $P = 0.007$, Sham vs. TAA + Saline: $P = 0.012$, Sham vs. TAA + MitoQ: $P = 0.003$, TAA + Saline vs. BDL + MitoQ: $P = 0.517$.
Statistical analysis of body temperature 4 day in Sham, TAA + Saline, and TAA + MitoQ groups $F (2, 15) = 13.835$, $P < 0.001$, Sham vs. TAA + Saline: $P < 0.001$, Sham vs. TAA + MitoQ: $P = 0.002$, TAA + Saline vs. BDL + MitoQ: $P = 0.230$.
Statistical analysis of body temperature 5 day in Sham, TAA + Saline, and TAA + MitoQ groups $F (2, 15) = 15.722$, $P < 0.001$, Sham vs. TAA + Saline: $P < 0.001$, Sham vs. TAA + MitoQ: $P = 0.009$, TAA + Saline vs. BDL + MitoQ: $P = 0.010$.
Statistical analysis of body temperature 6 day in Sham, TAA + Saline, and TAA + MitoQ groups $F (2, 15) = 26.710$, $P < 0.001$, Sham vs. TAA + Saline: $P < 0.001$, Sham vs. TAA + MitoQ: $P = 0.013$, TAA + Saline vs. BDL + MitoQ: $P = 0.001$.

h Average State III

Statistical analysis of average state III in Sham, TAA + Saline, TAA + *Ucp2* OE, and TAA + MitoQ groups F (3, 12) = 38.548, *P* < 0.001, Sham vs. TAA + Saline: *P* < 0.001, TAA + Saline vs. TAA + *Ucp2* OE: *P* = 0.007, TAA + Saline vs. TAA + MitoQ: *P* = 0.019.

i Average State IV

Statistical analysis of average state IV in Sham, TAA + Saline, TAA + *Ucp2* OE, and TAA + MitoQ groups F (3, 12) = 1.057, *P* = 0.403, Sham vs. TAA + Saline: *P* = 0.187, TAA + Saline vs. TAA + *Ucp2* OE: *P* = 0.830, TAA + Saline vs. TAA + MitoQ: *P* = 0.535.

j RCR

Statistical analysis of RCR in Sham, TAA + Saline, TAA + *Ucp2* OE, and TAA + MitoQ groups F (3, 12) = 25.938, *P* < 0.001, Sham vs. TAA + Saline: *P* < 0.001, TAA + Saline vs. TAA + *Ucp2* OE: *P* = 0.049, TAA + Saline vs. TAA + MitoQ: *P* = 0.037.

k Complex III

Statistical analysis of complex III in Sham, TAA + Saline, TAA + *Ucp2* OE, and TAA + MitoQ groups F (3, 16) = 11.924, *P* < 0.001, Sham vs. TAA + Saline: *P* < 0.001, TAA + Saline vs. TAA + *Ucp2* OE: *P* = 0.019, TAA + Saline vs. TAA + MitoQ: *P* = 0.007.

l Complex IV

Statistical analysis of complex IV in Sham, TAA + Saline, TAA + *Ucp2* OE, and TAA + MitoQ groups F (3, 16) = 16.0561, *P* < 0.0001, Sham vs. TAA + Saline: *P* < 0.001, TAA + Saline vs. TAA + *Ucp2* OE: *P* = 0.001, TAA + Saline vs. TAA + MitoQ: *P* = 0.003.

m ATP

Statistical analysis of ATP in Sham, TAA + Saline, TAA + *Ucp2* OE, and TAA + MitoQ groups F (3, 16) = 8.520, *P* = 0.001, Sham vs. TAA + Saline: *P* < 0.001, TAA + Saline vs. TAA + *Ucp2* OE: *P* = 0.859, TAA + Saline vs. TAA + MitoQ: *P* = 0.032.

Supplementary Fig.1

Two-tailed, unpaired, Student's *t* -test for d and i

Two-tailed, paired, Student's *t* -test for f, g, k and l.

One-way ANOVA with LSD's multiple comparison tests for c, d, and e.

c – OFT-Total distance:

Statistical analysis of total distance in Sham, TAA 100 mg/kg, TAA 150 mg/kg and, TAA 200 mg/kg groups F (3, 15) = 7.786, *P* = 0.002, Sham vs. TAA 100 mg/kg: *P* = 0.934, Sham vs. TAA 150 mg/kg *P* = 0.0102, and Sham vs.TAA 200 mg/kg *P* = 0.011.

d - OFT-Average speed:

Statistical analysis of average speed in Sham, TAA 100 mg/kg, TAA 150 mg/kg and, TAA 200 mg/kg groups F (3, 15) = 6.751, *P* = 0.004, Sham vs. TAA 100 mg/kg: *P* = 0.803, Sham vs. TAA 150 mg/kg *P* = 0.021, and Sham vs.TAA 200 mg/kg *P* = 0.009.

e – Rotarod:

Statistical analysis of duration in Sham, TAA 100 mg/kg, TAA 150 mg/kg and, TAA 200 mg/kg groups F (3, 15) = 9.877, *P* < 0.001, Sham vs. TAA 100 mg/kg: *P* = 0.336, Sham vs. TAA 150 mg/kg *P* = 0.022, and Sham vs.TAA 200 mg/kg *P* < 0.001.

Supplementary Fig.2

One-way ANOVA with LSD's multiple comparison tests was used.

Statistical analysis of brain water content in Sham, TAA, and MitoQ groups F (2, 12) = 7.958, *P* = 0.006, Sham vs. TAA: *P* = 0.002, TAA vs. MitoQ: *P* = 0.038.

Supplementary Fig.3

One-way ANOVA with LSD's multiple comparison tests for all data.

b-ALT:

Statistical analysis of ALT in Control, MitoQ, *Ucp2* NC, *Ucp2* KD and *Ucp2* OE groups F (4, 20) = 1.5240, *P* = 0.233, Control vs. MitoQ: *P* = 1.000, Control vs. *Ucp2* NC *P* = 1.000, Control vs. *Ucp2* KD *P* = 0.508, Control vs. *Ucp2* OE *P* = 0.557.

C-AST:

Statistical analysis of AST in Control, MitoQ, *Ucp2* NC, *Ucp2* KD and *Ucp*2 OE groups F (4, 20) = 1.6330, *P* = 0.205, Control vs. MitoQ: *P* = 1.000, Control vs. *Ucp2* NC *P* = 1.000, Control vs. *Ucp2* KD *P* = 0.452, Control vs. *Ucp2* OE *P* = 0.464.

D-Rotarod:

Statistical analysis of Duration in Control, MitoQ, *Ucp2* NC, *Ucp2* KD and *Ucp2* OE groups F (4, 20) = 1.667, *P* = 0.197, Control vs. MitoQ: *P* = 0.828, Control vs. *Ucp2* NC *P* = 0.741, Control vs. *Ucp2* KD *P* = 0.833, Control vs. *Ucp2* OE *P* = 1.000.

F

OFT-Total distance:

Statistical analysis of total distance in Control, MitoQ, *Ucp2* NC, *Ucp2* KD and *Ucp2* OE groups F (4, 20) = 0.224, *P* = 0.922, Control vs. MitoQ: *P* = 0.999, Control vs. *Ucp2* NC *P* = 0.993, Control vs. *Ucp2* KD *P* = 1.000, Control vs. *Ucp2* OE *P* = 0.974.

OFT-Average speed:

Statistical analysis of average speed in Control, MitoQ, *Ucp2* NC, *Ucp2* KD and *Ucp2* OE groups F (4, 20) = 0.224, *P* = 0.922, Control vs. MitoQ: *P* = 0.999, Control vs. *Ucp2* NC *P* = 0.993, Control vs. *Ucp2* KD *P* = 1.000, Control vs. *Ucp2* OE *P* = 0.922.

OFT-Number of activities:
Statistical analysis of number of activities in Control, MitoQ, *Ucp2* NC, *Ucp2* KD and *Ucp2* OE groups F (4, 20) = 0.829, $P = 0.522$, Control vs. MitoQ: $P = 0.921$, Control vs. *Ucp2* NC $P = 1.000$, Control vs. *Ucp2* KD $P = 1.000$, Control vs. Ucp2 OE $P = 0.855$.

Supplementary Fig.4

One-way ANOVA with LSD's multiple comparison tests for b.

b-Nissl

Statistical analysis of brain water content in Sham, TAA + Saline, and TAA + MitoQ groups F (2, 6) = 0.467, $P = 0.648$, Sham vs. TAA: $P = 0.885$, TAA vs. TAA + MitoQ: $P = 0.402$.

Supplementary Fig.5

One-way ANOVA with LSD's multiple comparison tests for all data.

c

OFT-Total distance:

one-way ANOVA with LSD's multiple comparison tests for all.

Statistical analysis of Total distance in mCherry + DCZ + TAA, Gi + DCZ + TAA and Gq + DCZ + TAA groups F (2, 12) = 43.230, $P < 0.001$, mCherry + DCZ + TAA vs. Gi + DCZ + TAA: $P < 0.001$, mCherry + DCZ + TAA vs. Gq + DCZ + TAA: $P = 0.026$.

OFT-Average speed:

Statistical analysis of average speed in mCherry + DCZ + TAA, Gi + DCZ + TAA and Gq + DCZ + TAA groups F (2, 12) = 54.501, $P < 0.001$, mCherry + DCZ + TAA vs. Gi + DCZ + TAA: $P < 0.001$, mCherry + DCZ + TAA vs. Gq + DCZ + TAA: $P = 0.028$.

OFT- activities number:

Statistical analysis of activities number in mCherry + DCZ + TAA, Gi + DCZ + TAA and Gq + DCZ + TAA groups F (2, 12) = 14.531, $P < 0.001$, mCherry + DCZ + TAA vs. Gi + DCZ + TAA: $P = 0.008$, mCherry + DCZ + TAA vs. Gq + DCZ + TAA: $P = 0.049$.

OFT- activities time:

Statistical analysis of activities time in mCherry + DCZ + TAA, Gi + DCZ + TAA and Gq + DCZ + TAA groups F (2, 12) = 41.554, $P < 0.001$, mCherry + DCZ + TAA vs. Gi + DCZ + TAA: $P < 0.001$, mCherry + DCZ + TAA vs. Gq + DCZ + TAA: $P = 0.046$.

d

Rotarod:

Statistical analysis of duration in mCherry + DCZ + TAA, Gi + DCZ + TAA and Gq + DCZ + TAA groups F (2, 12) = 16.7314, $P = 0.0003$, mCherry + DCZ + TAA vs. Gi + DCZ + TAA: $P = 0.0048$, mCherry + DCZ + TAA vs. Gq + DCZ + TAA: $P = 0.0408$.

Supplementary Fig.6

One-way ANOVA with LSD's multiple comparison tests for all data.

a

SOD1:

one-way ANOVA with LSD's multiple comparison tests for all.

Statistical analysis of SOD1 in mCherry + CNO, Gi + CNO and Gq + CNO groups F (2, 6) = 1.340, *P* = 0.330, mCherry + CNO vs. Gi + CNO: *P* = 0.232, mCherry + CNO vs. Gq + CNO: *P* = 0.187.

GPX1:

Statistical analysis of GPX1 in mCherry + CNO, Gi + CNO and Gq + CNO groups F (2, 6) = 1.758, *P* = 0.251, mCherry + CNO vs. Gi + CNO: *P* = 0.113, mCherry + CNO vs. Gq + CNO: *P* = 0.522.

b

LC3B:

Statistical analysis of LC3B in mCherry + CNO, Gi + CNO and Gq + CNO groups F (2, 6) = 2.736, *P* = 0.143, mCherry + CNO vs. Gi + CNO: *P* = 0.163, mCherry + CNO vs. Gq + CNO: *P* = 0.514.

PINK1:

Statistical analysis of PINK1 in mCherry + CNO, Gi + CNO and Gq + CNO groups F (2, 6) = 1.0130, *P* = 0.418, mCherry + CNO vs. Gi + CNO: *P* = 0.237, mCherry + CNO vs. Gq + CNO: *P* = 0.865.
