# Supplementary material for: Effects of oxidative stress on hepatic encephalopathy pathogenesis in mice
Source: Nat Commun. 2023 Jul 24;14:4456. doi: 10.1038/s41467-023-40081-8 (PMC10366183; doi:10.1038/s41467-023-40081-8)
Supplement: Supplementary file 2 — Reporting Summary [file 41467_2023_40081_MOESM2_ESM.pdf]

nature portfolio

Corresponding author(s): Yanling Yang
Yayun Wang
Yong Chen

Last updated by author(s): Jun 8, 2023

Reporting Summary

Nature Portfolio wishes to improve the reproducibility of the work that we publish. This form provides structure for consistency and transparency in reporting. For further information on Nature Portfolio policies, see our Editorial Policies and the Editorial Policy Checklist.

Statistics

For all statistical analyses, confirm that the following items are present in the figure legend, table legend, main text, or Methods section.

n/a	Confirmed	
☐	☒	The exact sample size (*n*) for each experimental group/condition, given as a discrete number and unit of measurement
☐	☒	A statement on whether measurements were taken from distinct samples or whether the same sample was measured repeatedly
☐	☒	The statistical test(s) used AND whether they are one- or two-sided<br>*Only common tests should be described solely by name; describe more complex techniques in the Methods section.*
☐	☒	A description of all covariates tested
☐	☒	A description of any assumptions or corrections, such as tests of normality and adjustment for multiple comparisons
☐	☒	A full description of the statistical parameters including central tendency (e.g. means) or other basic estimates (e.g. regression coefficient) AND variation (e.g. standard deviation) or associated estimates of uncertainty (e.g. confidence intervals)
☐	☒	For null hypothesis testing, the test statistic (e.g. *F*, *t*, *r*) with confidence intervals, effect sizes, degrees of freedom and *P* value noted<br>*Give P values as exact values whenever suitable.*
☒	☐	For Bayesian analysis, information on the choice of priors and Markov chain Monte Carlo settings
☒	☐	For hierarchical and complex designs, identification of the appropriate level for tests and full reporting of outcomes
☒	☐	Estimates of effect sizes (e.g. Cohen's *d*, Pearson's *r*), indicating how they were calculated

Our web collection on statistics for biologists contains articles on many of the points above.

Software and code

Policy information about availability of computer code

Data collection	Confocal imaging (Olympus System FV1000). Clampex software (v.10.02, Axon Instruments) for whole cell patch-clamp recordings; Video system from Shanghai Mobile Datum was used in open field test. Motor defect and coordination were analyzed using the Rotarod test (BYZ-007, China) and CatWalk test (Noduls). The respiratory energy metabolism monitoring system (Coulumbus Oxymax / CLAMS). Mitochondria Respiration was measured by the CLARIO star plus instrument (BMG LABTECH). ImageJ Fiji software was used for image counting and fluorescence intensity.
Data analysis	Imaging data were analyzed with FV10-ASW 4.2 Viewer and Image J Fiji. Electrophysiology data were analyzed by Mini Analysis (v.6.0, Synaptosoft Inc) and Clampfit (v.10.7, Axon Instruments). Data were analyzed using the SPSS software version (v.26.0) and GraphPad Prism (v.8.02).

For manuscripts utilizing custom algorithms or software that are central to the research but not yet described in published literature, software must be made available to editors and reviewers. We strongly encourage code deposition in a community repository (e.g. GitHub). See the Nature Portfolio guidelines for submitting code & software for further information.

Data

Policy information about availability of data

All manuscripts must include a data availability statement. This statement should provide the following information, where applicable:

- Accession codes, unique identifiers, or web links for publicly available datasets
- A description of any restrictions on data availability
- For clinical datasets or third party data, please ensure that the statement adheres to our policy

The datasets generated during and/or analysed during the current study are available from the corresponding author on reasonable request.

Human research participants

Policy information about studies involving human research participants and Sex and Gender in Research.

Reporting on sex and gender	N/A.
Population characteristics	N/A.
Recruitment	N/A.
Ethics oversight	N/A.

Note that full information on the approval of the study protocol must also be provided in the manuscript.

Field-specific reporting

Please select the one below that is the best fit for your research. If you are not sure, read the appropriate sections before making your selection.

☒ Life sciences ☐ Behavioural & social sciences ☐ Ecological, evolutionary & environmental sciences

For a reference copy of the document with all sections, see nature.com/documents/nr-reporting-summary-flat.pdf

Life sciences study design

All studies must disclose on these points even when the disclosure is negative.

Sample size	Based on the literature and our previous studies (PMID: 29857139; PMID: 34890554), we chose the sample size routinely used for animal experiments, western blot, immunofluorescence analysis, respiration of isolated mitochondria and ELISA.
Data exclusions	In behavior tests, all data are excluded when injections sites miss the target area. Mice in poor condition and incapacitated are excluded from the experiments.
Replication	Each experiment is repeated for at least two times to verify the reproducibility of experimental findings.
Randomization	The mice are randomized to put into separate groups for allocation.
Blinding	Double-blind data collection and analysis are used in this study.

Behavioural & social sciences study design

All studies must disclose on these points even when the disclosure is negative.

Study description	
Research sample	
Sampling strategy	
Data collection	
Timing	

Data exclusions

Non-participation

Randomization

Ecological, evolutionary & environmental sciences study design

All studies must disclose on these points even when the disclosure is negative.

Study description

Research sample

Sampling strategy

Data collection

Timing and spatial scale

Data exclusions

Reproducibility

Randomization

Blinding

Did the study involve field work? ☐ Yes ☒ No

Field work, collection and transport

Field conditions

Location

Access & import/export

Disturbance

Reporting for specific materials, systems and methods

We require information from authors about some types of materials, experimental systems and methods used in many studies. Here, indicate whether each material, system or method listed is relevant to your study. If you are not sure if a list item applies to your research, read the appropriate section before selecting a response.

Materials & experimental systems

n/a	Involved in the study	
☐	☒	Antibodies
☒	☐	Eukaryotic cell lines
☒	☐	Palaeontology and archaeology
☐	☒	Animals and other organisms
☒	☐	Clinical data
☒	☐	Dual use research of concern

Methods

n/a	Involved in the study	
☒	☐	ChIP-seq
☒	☐	Flow cytometry
☒	☐	MRI-based neuroimaging

Antibodies

Antibodies used: primary antibody used:

Antibodies used

Mouse polyclonal anti-SOD1(1:1000, Cat. #A0274, Abclonal, CN)
Mouse monoclonal anti-GPX1(1:1000, Cat. #A11166, Abclonal)
Rabbit polyclonal anti-UCP2(1:1000, Cat. #89326, Cell Signaling Technology, UAS)
Mouse monoclonal anti-UCP4 (1:1000, Cat. #sc-365295,Santa Cruz Biotechnology,USA)
Rabbit polyclonal anti-UCP5 (1:1000, Cat . #A13731, Abclonal)
Rabbit monoclonal anti-LC3B (1:1000, Cat. #3868, Cell Signaling Technology, UAS)
Rabbit polyclonal anti-PINK1 (1:1000, Cat. #ab23707,Abcam,MA,UK)
Rabbit monoclonal anti-DRP1 (1:1000, Cat. #8570s, Cell Signaling Technology)
Phospho-Ser616 DRP1 (1:1000, Cat. #3455, Cell Signaling Technology, UAS)
Rabbit monoclonal anti-MFN2 (1:1000, Cat. #9482S, Cell Signaling Technology, UAS)
Rabbit polyclonal anti-FIS1 (1:1000, Cat. # A19666, Abclonal)
Rabbit monoclonal anti-Mff (1:1000, Cat. # 84580s, Cell Signaling Technology, UAS)
Mouse anti-β-ACTIN (1:5000, Cat. # AC004, Abclonal)
Mouse monoclonal anti-GAD67 (1:100, Cat. #ab26116 ,Abcam)
Rabbit monoclonal anti-GAD65 (1:50, Cat. #5843s, Cell Signaling Technology, UAS)
Rabbit monoclonal anti-Parvalbumin (1:200, Cat. #80561s, Cell Signaling Technology, UAS)
Rabbit polyclonal anti-vGlut1 (1:500, Cat. #a12879, Abclonal)
Rabbit polyclonal anti-GFAP (1:200, Cat. #12389s, Cell Signaling Technology, UAS)
Rabbit monoclonal anti-NeuN (1:50, Cat. #ab190565, Abcam)
Secondary antibodies used:
HRP-Goat anti-Rabbit (1:5000, Cat. #A21020 Abbkine,CN)
HRP-Goat anti-Mouse (1:5000, Cat. #A21010 Abbkine)
Alexa 488-Goat anti-Rabbit (1:500, Cat. #A23220, Abbkine)
Alexa 488-Goat anti-Mouse (1:500, Cat.#A23210, Abbkine)
Alexa 594-Goat anti-Rabbit (1:500, Cat. #A23420, Abbkine)
Alexa 594-Goat anti-Mouse (1:500, Cat. #A23410, Abbkine)
Tertiary antibodies used:
DAPI (1:500, Cat. #BMD0063, Abbkine)

Validation

The specificity and application of all the antibodies are validated by companies
Mouse anti-SOD1
Validation:https://abclonal.com.cn/catalog/A0274
Mouse anti-GPX1
Validation:https://abclonal.com.cn/catalog/A11166
Rabbit anti-UCP2
Validation:https://www.cellsignal.cn/products/primary-antibodies/ucp2-d1o5v-rabbit-mab/89326
Mouse anti-UCP4
Validation:https://www.scbt.com/zh/p/ucp4-antibody-a-5
Rabbit anti-UCP5
Validation:https://abclonal.com.cn/catalog/A13731
Rabbit anti-LC3B
Validation:https://www.cellsignal.cn/products/primary-antibodies/lc3b-d11-xp-rabbit-mab/3868Rabbit
Rabbit anti-PINK1
Validation:https://www.abcam.cn/products/primary-antibodies/pink1-antibody-ab23707.html
Rabbit anti-DRP1
Validation:https://www.cellsignal.cn/products/primary-antibodies/drp1-d6c7-rabbit-mab/8570
Phospho-Ser616 DRP1
Validation:https://www.cellsignal.cn/products/primary-antibodies/phospho-drp1-ser616-antibody/3455
Rabbit anti-MFN2
Validation:https://www.cellsignal.cn/products/primary-antibodies/mitofusin-2-d2d10-rabbit-mab/9482
Rabbit anti-FIS1
Validation:https://abclonal.com.cn/catalog/A19666
Rabbit anti-MFF
Validation:https://www.cellsignal.cn/products/primary-antibodies/mff-e5w4m-xp-rabbit-mab/84580
Mouse anti-β-ACTIN
Validation:https://abclonal.com.cn/catalog/AC004
Mouse anti-GAD(67)1
Validation:https://www.abcam.cn/products/primary-antibodies/gad67-antibody-k-87-ab26116.html
Rabbit anti-GAD(65)2
Validation:https://www.cellsignal.cn/products/primary-antibodies/gad2-d5g2-xp-rabbit-mab/5843
Rabbit anti-Parvalbumin
Validation:https://www.cellsignal.cn/products/primary-antibodies/parvalbumin-e8n2u-xp-rabbit-mab/80561
Rabbit anti-vGlut1
Validation:https://abclonal.com.cn/catalog/A12879
Rabbit anti-GFAP
Validation:https://www.cellsignal.cn/products/primary-antibodies/gfap-d1f4q-xp-rabbit-mab/12389
Rabbitanti-NeuN
Validation:https://www.abcam.cn/products/primary-antibodies/alexa-fluor-647-neun-antibody-epr12763-neuronal-marker-ab190565.html

Secondary antibodies used:
HRP-Goat anti-Rabbit
Validation:https://www.abbkine.cn/product/a21020/
HRP-Goat anti-Mouse
Validation:https://www.abbkine.cn/product/a21010/
Alexa 488-Goat anti-Rabbit

Validation:https://www.abbkine.cn/product/a23220/
Alexa 488-Goat anti-Mouse
Validation:https://www.abbkine.cn/product/a23210/
Alexa 594-Goat anti-Rabbit
Validation:https://www.abbkine.cn/product/a23420/
Alexa 594-Goat anti-Mouse
Validation:https://www.abbkine.cn/product/a23410/

Tertiary antibodies used:
DAPI
https://www.abbkine.cn/product/bmd0063/

Eukaryotic cell lines

Policy information about cell lines and Sex and Gender in Research

Cell line source(s)	
Authentication	
Mycoplasma contamination	
Commonly misidentified lines (See ICLAC register)	

Palaeontology and Archaeology

Specimen provenance	
Specimen deposition	
Dating methods	

☐ Tick this box to confirm that the raw and calibrated dates are available in the paper or in Supplementary Information.

Ethics oversight	

Note that full information on the approval of the study protocol must also be provided in the manuscript.

Animals and other research organisms

Policy information about studies involving animals; ARRIVE guidelines recommended for reporting animal research, and Sex and Gender in Research

Laboratory animals	We use adult male mice (6 - 8 w) C57BL/6J for TAA, and adult male mice (10 - 12 w) C57BL/6 for BDL, FOS-cre ERT2 (TRAP2), GAD2-ires-cre, GFP-Mito tagfloxed, and GAD2-Mito-GFP male mice. Ambient temperature (18 - 26?) and humidity (40 - 70%) have been controlled. All mice are housed in a 12 h light (8 am) / dark (8 pm) environment.
Wild animals	The study does not involve wild animals.
Reporting on sex	To avoid the effects of gender differences and hormone level fluctuations on animal behavioral performances, all experimental animals used in the present study are male mice.
Field-collected samples	The study does not involve samples collected from the field.
Ethics oversight	All experiments in this study are performed in accordance with the ethical guidelines of the International Association for the Study of Pain, and approved by the Pain Research Committee of the Fourth Military Medical University.

Note that full information on the approval of the study protocol must also be provided in the manuscript.

Clinical data

Policy information about clinical studies
All manuscripts should comply with the ICMJE guidelines for publication of clinical research and a completed CONSORT checklist must be included with all submissions.

Clinical trial registration	
Study protocol	

Data collection

Outcomes

Dual use research of concern

Policy information about dual use research of concern

Hazards

Could the accidental, deliberate or reckless misuse of agents or technologies generated in the work, or the application of information presented in the manuscript, pose a threat to:

No	Yes	
☒	☐	Public health
☒	☐	National security
☒	☐	Crops and/or livestock
☒	☐	Ecosystems
☒	☐	Any other significant area

Experiments of concern

Does the work involve any of these experiments of concern:

No	Yes	
☒	☐	Demonstrate how to render a vaccine ineffective
☒	☐	Confer resistance to therapeutically useful antibiotics or antiviral agents
☒	☐	Enhance the virulence of a pathogen or render a nonpathogen virulent
☒	☐	Increase transmissibility of a pathogen
☒	☐	Alter the host range of a pathogen
☒	☐	Enable evasion of diagnostic/detection modalities
☒	☐	Enable the weaponization of a biological agent or toxin
☒	☐	Any other potentially harmful combination of experiments and agents

ChIP-seq

Data deposition

☐ Confirm that both raw and final processed data have been deposited in a public database such as GEO.

☐ Confirm that you have deposited or provided access to graph files (e.g. BED files) for the called peaks.

Data access links
May remain private before publication.

Files in database submission

Genome browser session
(e.g. UCSC)

Methodology

Replicates

Sequencing depth

Antibodies

Peak calling parameters

Data quality

Software

Flow Cytometry

Plots

Confirm that:

- [ ] The axis labels state the marker and fluorochrome used (e.g. CD4-FITC).
- [ ] The axis scales are clearly visible. Include numbers along axes only for bottom left plot of group (a 'group' is an analysis of identical markers).
- [ ] All plots are contour plots with outliers or pseudocolor plots.
- [ ] A numerical value for number of cells or percentage (with statistics) is provided.

Methodology

Sample preparation

Instrument

Software

Cell population abundance

Gating strategy

- [ ] Tick this box to confirm that a figure exemplifying the gating strategy is provided in the Supplementary Information.

Magnetic resonance imaging

Experimental design

Design type

Design specifications

Behavioral performance measures

Acquisition

Imaging type(s)

Field strength

Sequence & imaging parameters

Area of acquisition

Diffusion MRI [ ] Used [x] Not used

Preprocessing

Preprocessing software

Normalization

Normalization template

Noise and artifact removal

Volume censoring

Statistical modeling & inference

Model type and settings

Effect(s) tested

Specify type of analysis: [ ] Whole brain [ ] ROI-based [ ] Both

Statistic type for inference
(See Eklund et al. 2016)

Correction

Models & analysis

n/a	Involved in the study
☐	☐ Functional and/or effective connectivity
☐	☐ Graph analysis
☐	☐ Multivariate modeling or predictive analysis

Functional and/or effective connectivity

Graph analysis

Multivariate modeling and predictive analysis
